# Supplementary material for: Doping strategy in metavalently bonded materials for advancing thermoelectric performance
Source: Nat Commun. 2024 Sep 27;15:8286. doi: 10.1038/s41467-024-52645-3 (PMC11436876; doi:10.1038/s41467-024-52645-3)
Supplement: Supplementary file 1 — Supplementary Information [file 41467_2024_52645_MOESM1_ESM.pdf]

Supporting Information

Doping Strategy in Metavalently Bonded Materials for Advancing Thermoelectric Performance

Ming Liu[1,2], Muchun Guo[3], Haiyan Lyu[2], Yingda Lai[1], Yuke Zhu[1], Fengkai Guo[1]*, Yueyang Yang[2], Kuai Yu[1], Xingyan Dong[1], Zihang Liu[1], Wei Cai[1], Matthias Wuttig[2,4]*, Yuan Yu[2]*, & Jiehe Sui[1]*

[1] National Key Laboratory for Precision Hot Processing of Metals, Harbin Institute of Technology, Harbin 150001, China

[2] Institute of Physics (IA), RWTH Aachen University, Aachen 52074, Germany

[3] School of Materials Science and Engineering, Xihua University, Chengdu 610039, China

[4] Green IT (PGI 10), Forschungszentrum Jülich GmbH, 52428 Jülich, Germany

*Corresponding author: fkguo@hit.edu.cn (F. G.); wuttig@physik.rwth-aachen.de (M. W.); yu@physik.rwth-aachen.de (Y. Y.); suijiehe@hit.edu.cn (J. S.)

Supplementary Text:

Average sound velocity (v) is calculated from the sound velocity as follows[1].

$$v=\left[\frac{1}{3}\left(\frac{1}{v_L^3}+\frac{2}{v_T^3}\right)\right]^{-1/3} \qquad (1)$$

Poisson ratio (r) is calculated by[1].

$$r=\frac{1-2(v_T/v_L)^2}{2-2(v_T/v_L)^2} \qquad (2)$$

Young's modulus (E) is calculated by[1].

$$E=\frac{\rho v_t^2(3v_L^2-4v_T^2)}{(v_L^2-v_T^2)} \qquad (3)$$

Grüneisen parameters (γ) is calculated by[1].

$$\gamma=\frac{3}{2}\frac{(1+r)}{(2-3r)} \qquad (4)$$

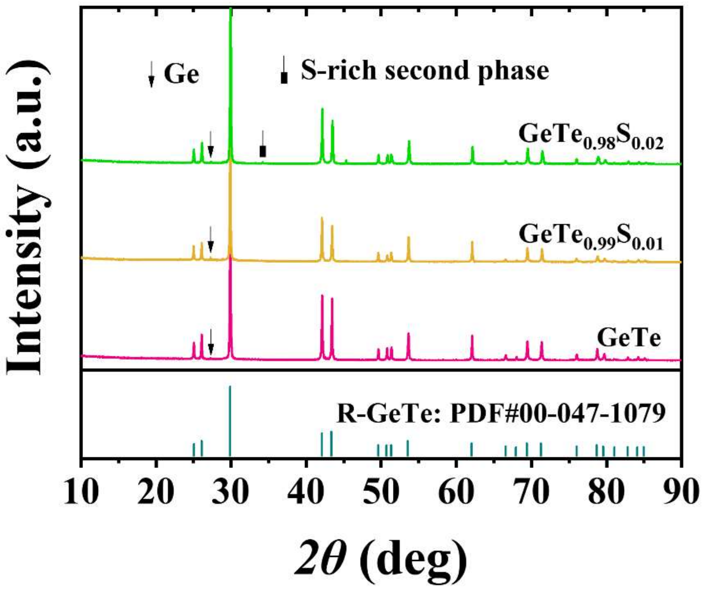

Fig. S1. Room-temperature XRD patterns of $GeTe_{1-x}S_x$ samples.

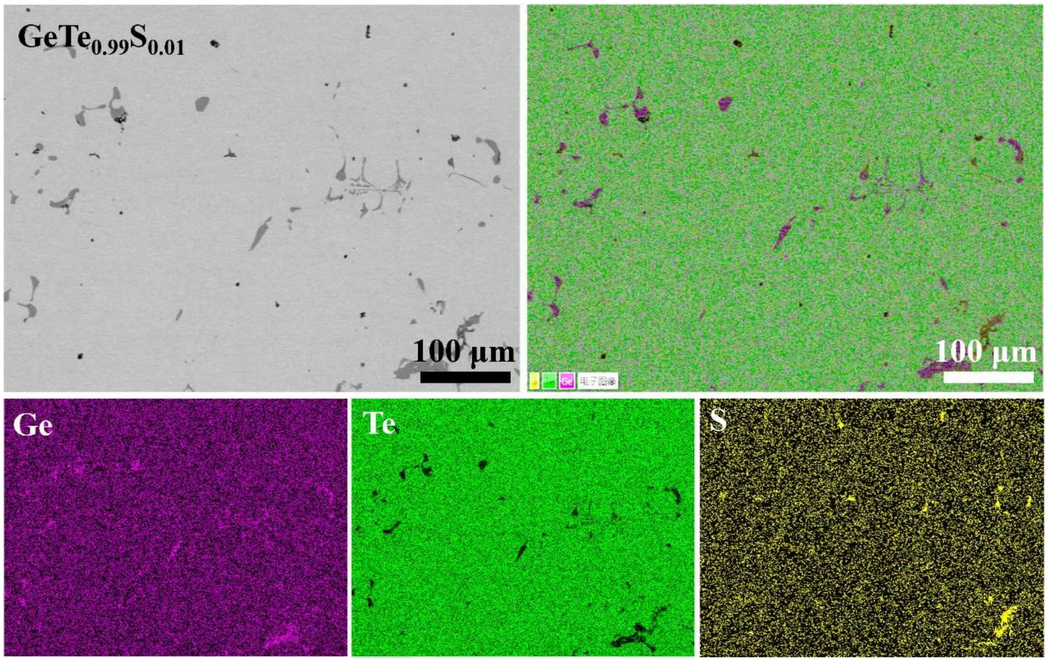

Fig. S2. BSE and EDS mapping of $GeTe_{0.99}S_{0.01}$ sample.

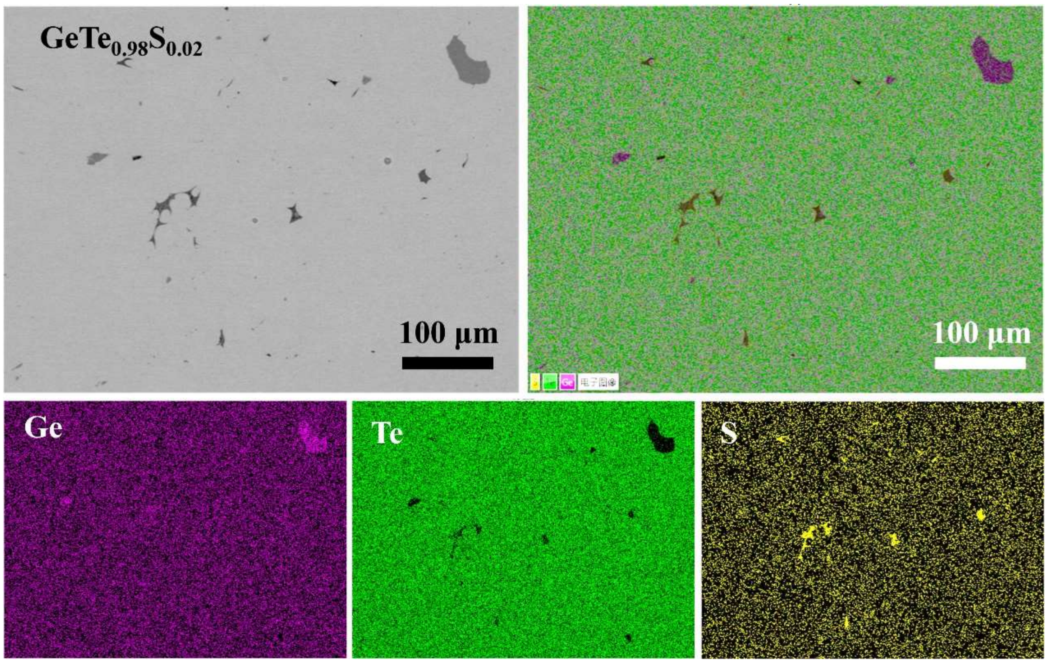

Fig. S3. BSE and EDS mapping of $GeTe_{0.98}S_{0.02}$ sample.

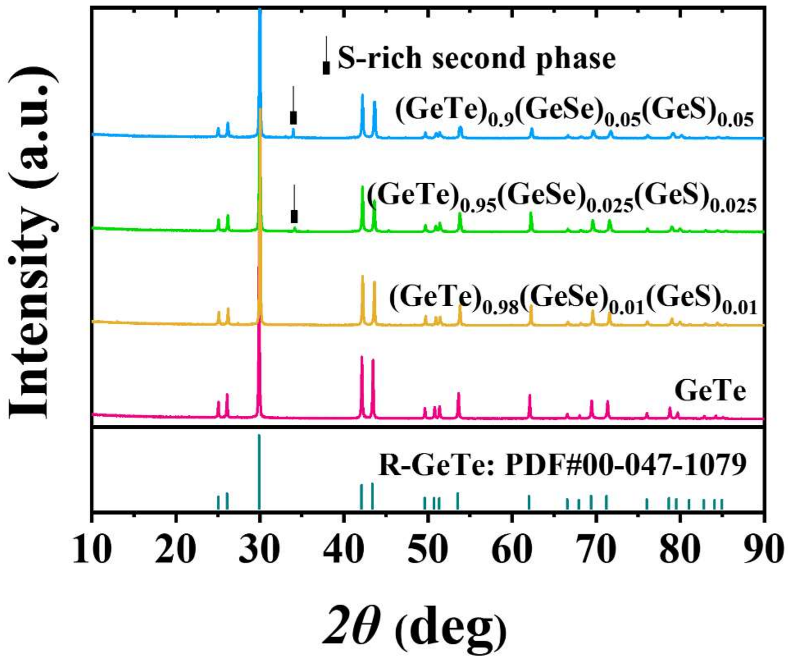

Fig. S4. Room-temperature XRD patterns of $(GeTe)_{1-2x}(GeSe)_x(GeS)_x$ samples.

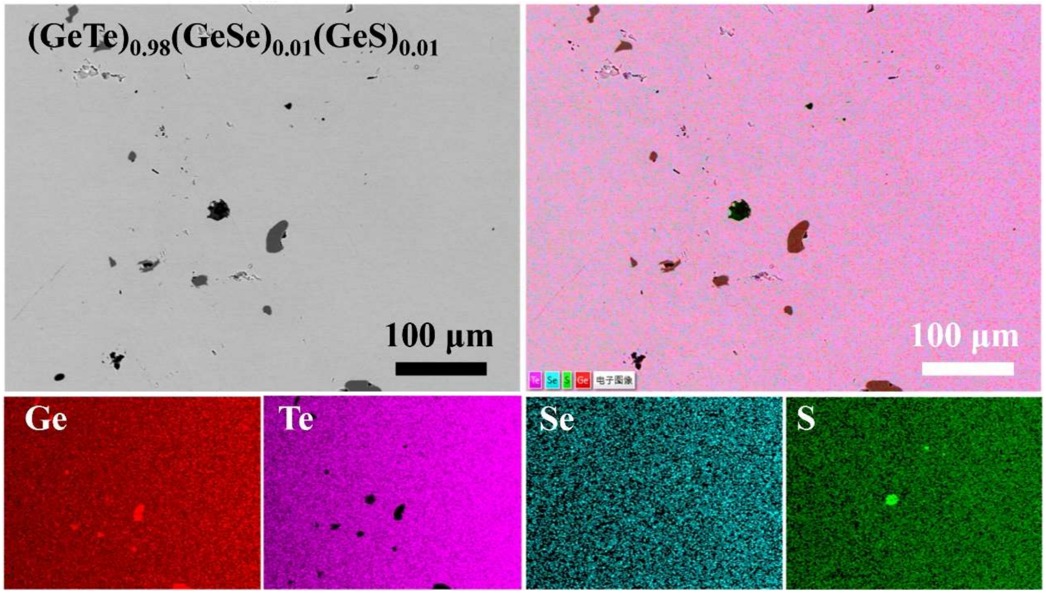

Fig. S5. BSE and EDS mapping of $(GeTe)_{0.98}(GeSe)_{0.01}(GeS)_{0.01}$ sample.

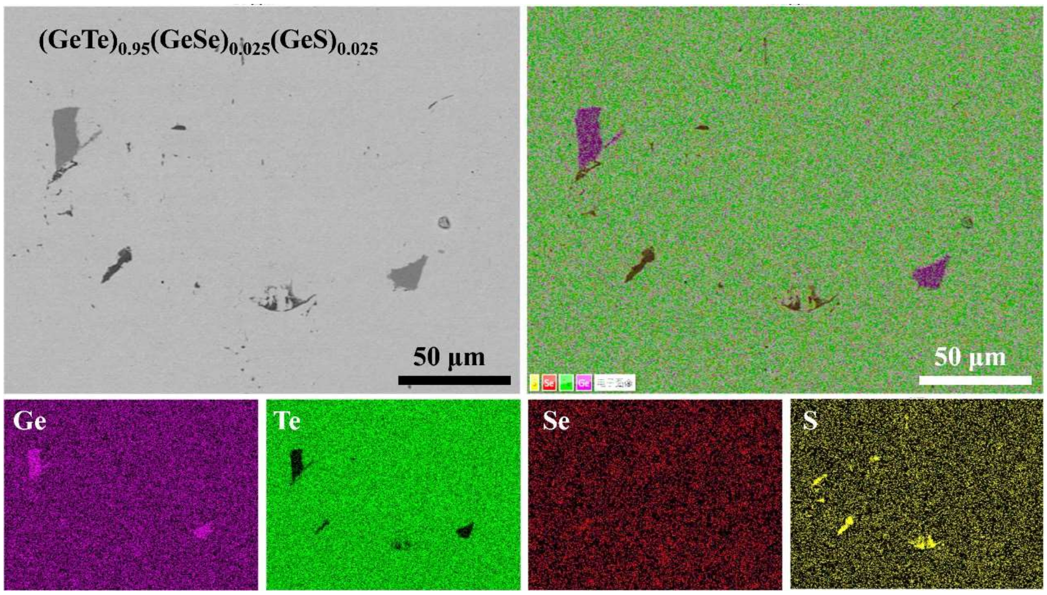

Fig. S6. BSE and EDS mapping of $(GeTe)_{0.95}(GeSe)_{0.025}(GeS)_{0.025}$ sample.

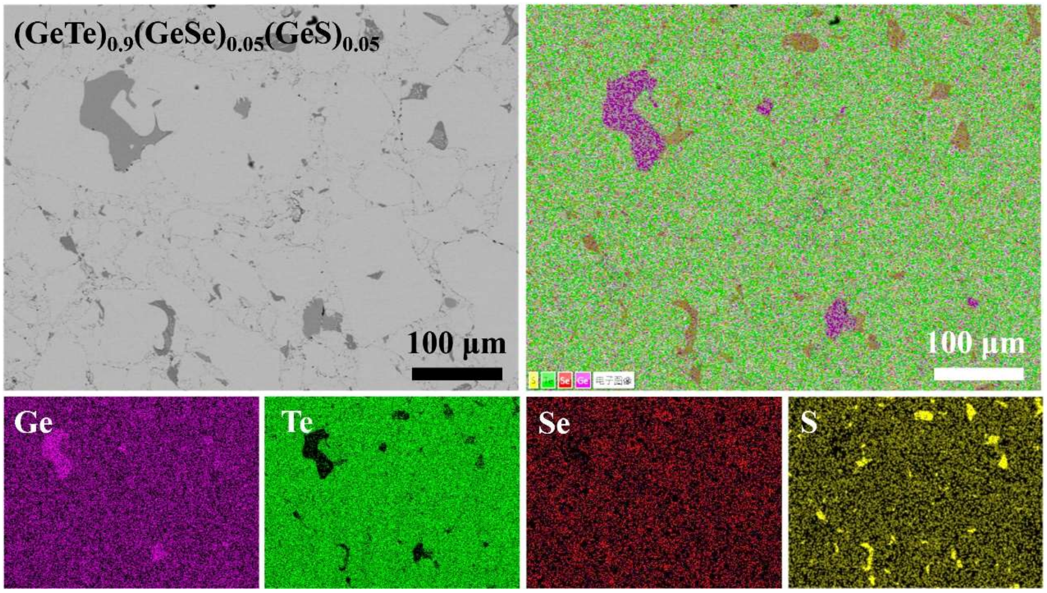

Fig. S7. BSE and EDS mapping of $(GeTe)_{0.9}(GeSe)_{0.05}(GeS)_{0.05}$ sample.

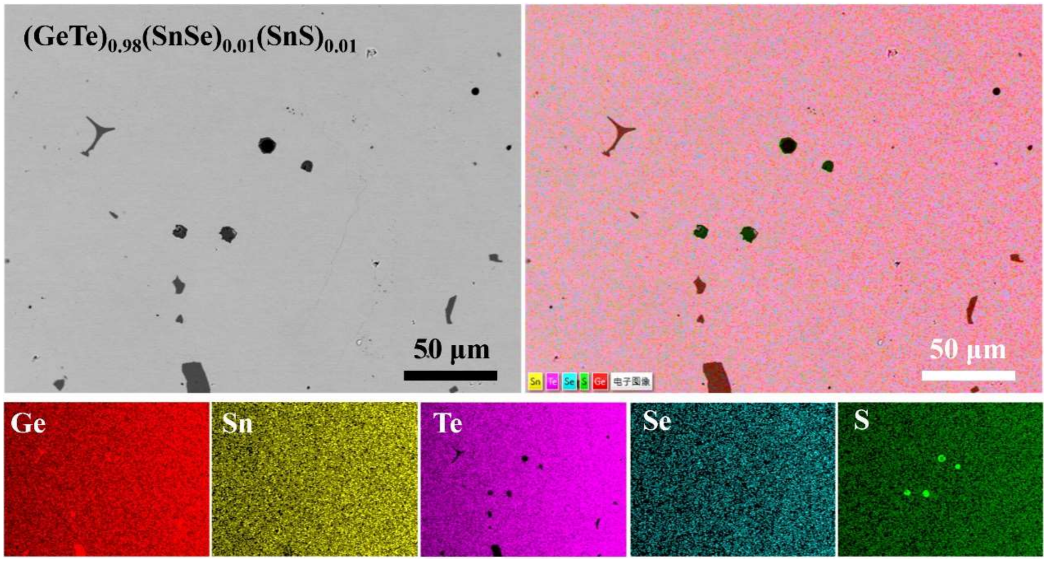

Fig. S8. BSE and EDS mapping of $(GeTe)_{0.98}(SnSe)_{0.01}(SnS)_{0.01}$ sample.

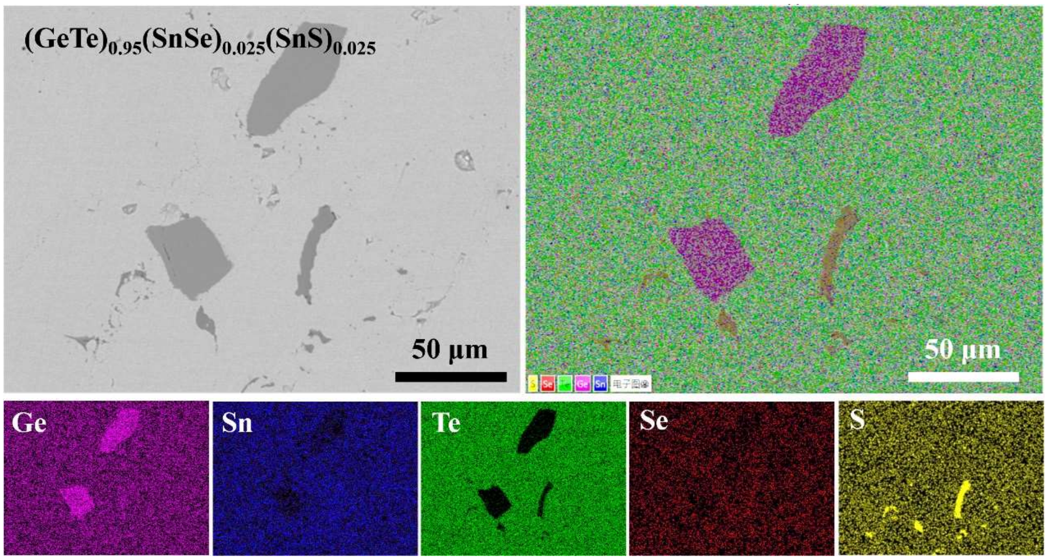

Fig. S9. BSE and EDS mapping of $(GeTe)_{0.95}(SnSe)_{0.025}(SnS)_{0.025}$ sample.

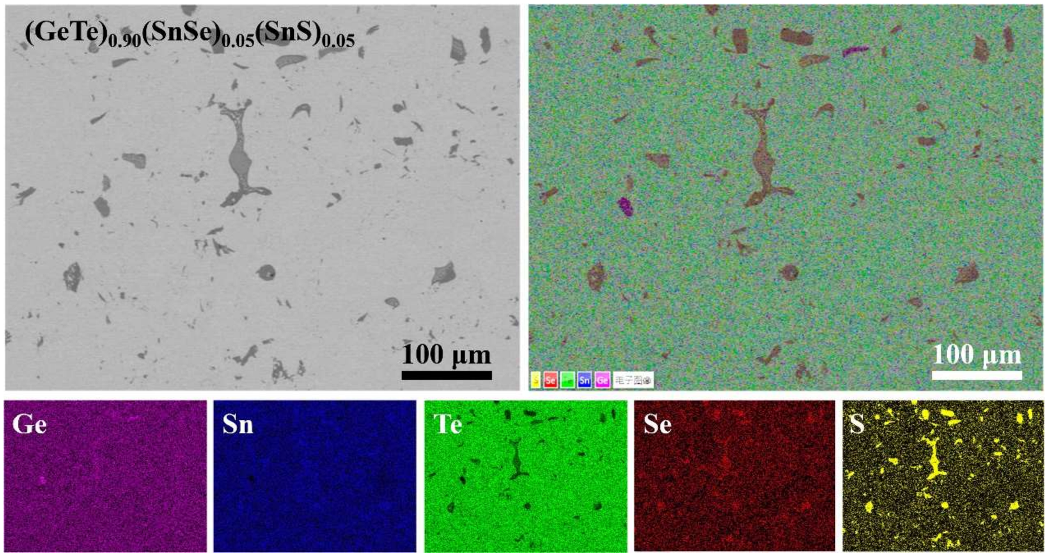

Fig. S10. BSE and EDS mapping of $(GeTe)_{0.9}(SnSe)_{0.05}(SnS)_{0.05}$ sample.

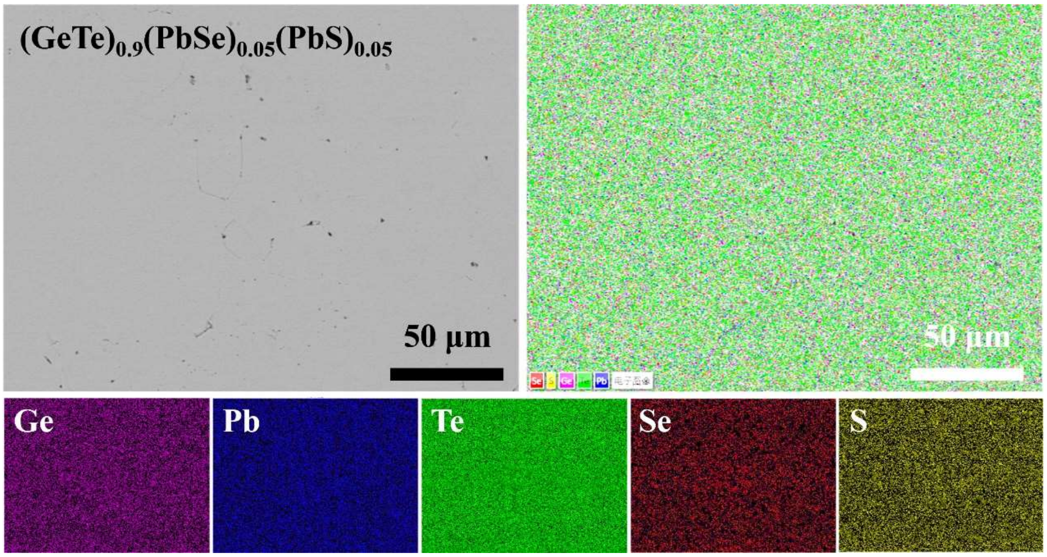

Fig. S11. BSE and EDS mapping of $(GeTe)_{0.9}(PbSe)_{0.05}(PbS)_{0.05}$ sample.

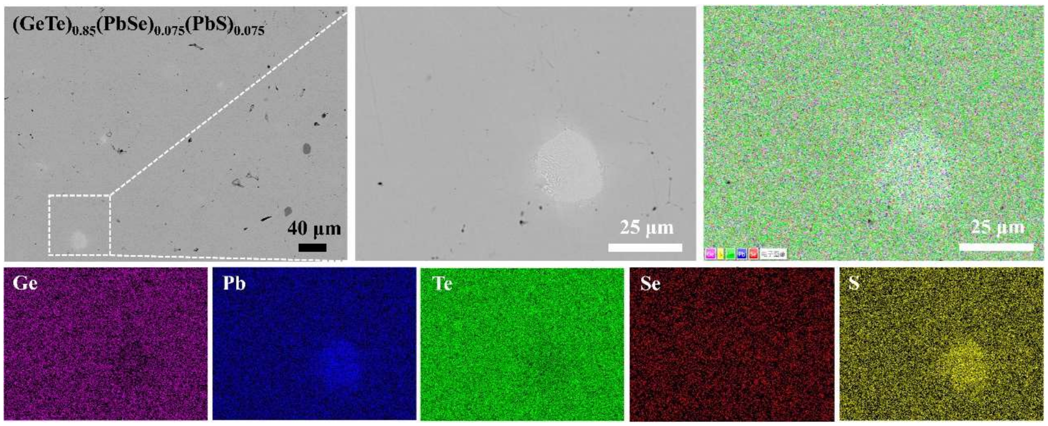

Fig. S12. BSE and EDS mapping of $(GeTe)_{0.85}(PbSe)_{0.075}(PbS)_{0.075}$ sample.

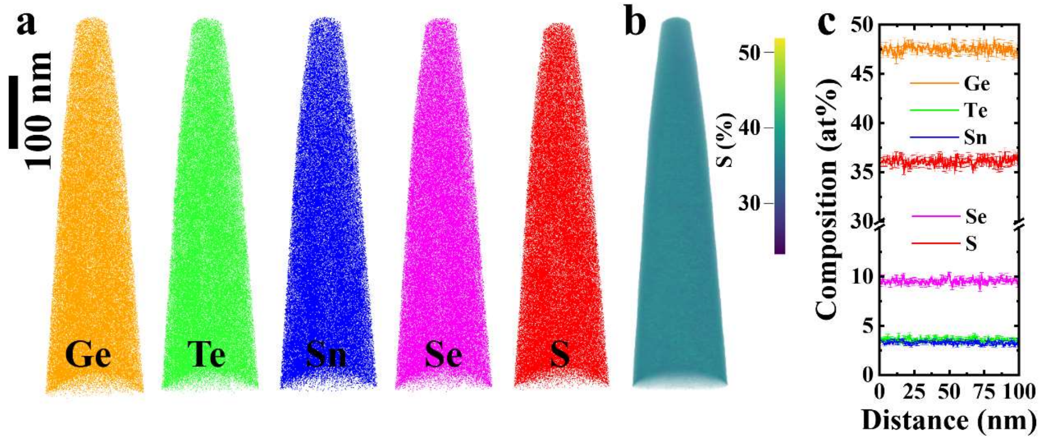

Fig. S13. **a** 3D reconstruction showing the distribution of Ge, Te, Sn, Se, and S, **b** volume rendering showing the composition of S in 3D space, **c** composition profile of elements taken from a cuboid region of interest along the vertical direction of the second phase of $(GeTe)_{0.9}(GeSe)_{0.05}(GeS)_{0.05}$ sample.

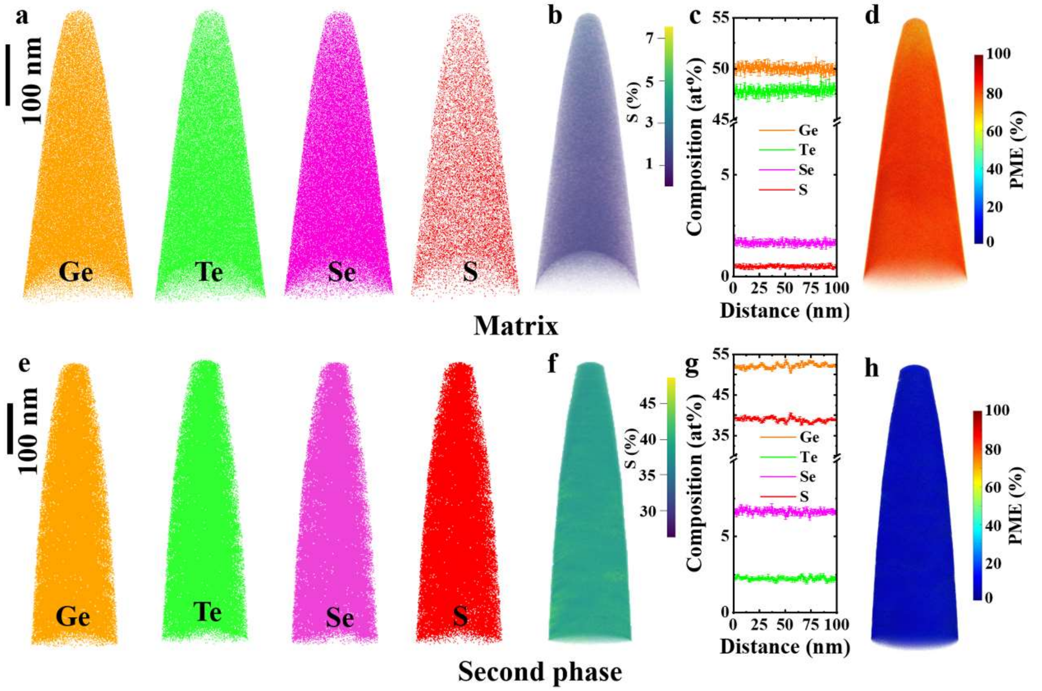

Fig. S14. **a** 3D reconstruction showing the distribution of Ge, Te, Se, and S, **b** volume rendering showing the composition of S in 3D space, **c** composition profile of elements taken from a cuboid region of interest along the vertical direction, **d** 3D PME map of the matrix of $(GeTe)_{0.9}(GeSe)_{0.05}(GeS)_{0.05}$ sample; **e** 3D reconstruction showing the distribution of Ge, Te, Se, and S, **f** volume rendering showing the composition of S in 3D space, **g** composition profile of elements taken from a cuboid region of interest along the vertical direction, **h** 3D PME map of the second phase of $(GeTe)_{0.9}(GeSe)_{0.05}(GeS)_{0.05}$ sample.

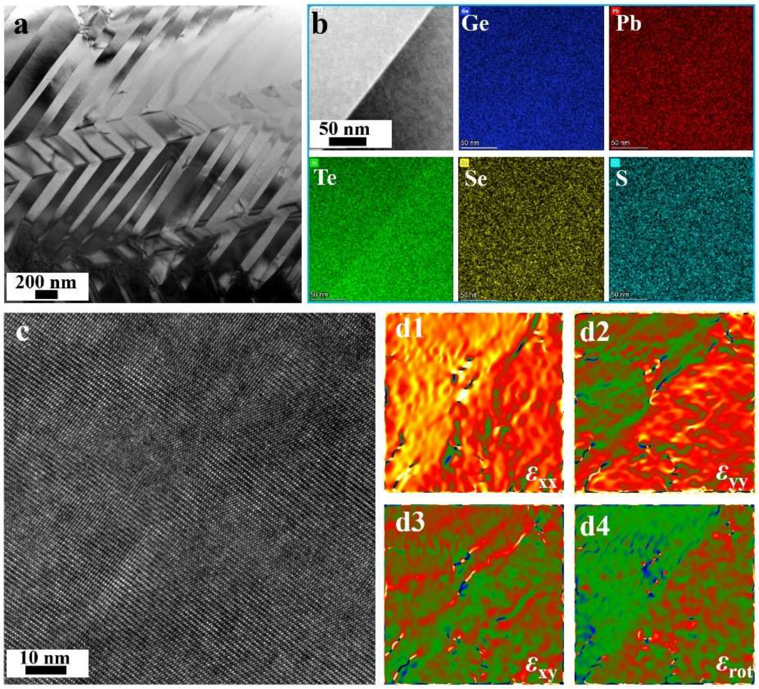

Fig. S15. **a** Transmission electron microscopy (TEM) image, **b** EDS mappings for $(GeTe)_{0.9}(PbSe)_{0.05}(PbS)_{0.05}$ sample, **c** high-magnification TEM image of domain boundary, **d1-d4** geometric phase analysis (GPA) strain maps along the ε_{xx}, ε_{yy}, ε_{xy}, and ε_{rot} of **c**.

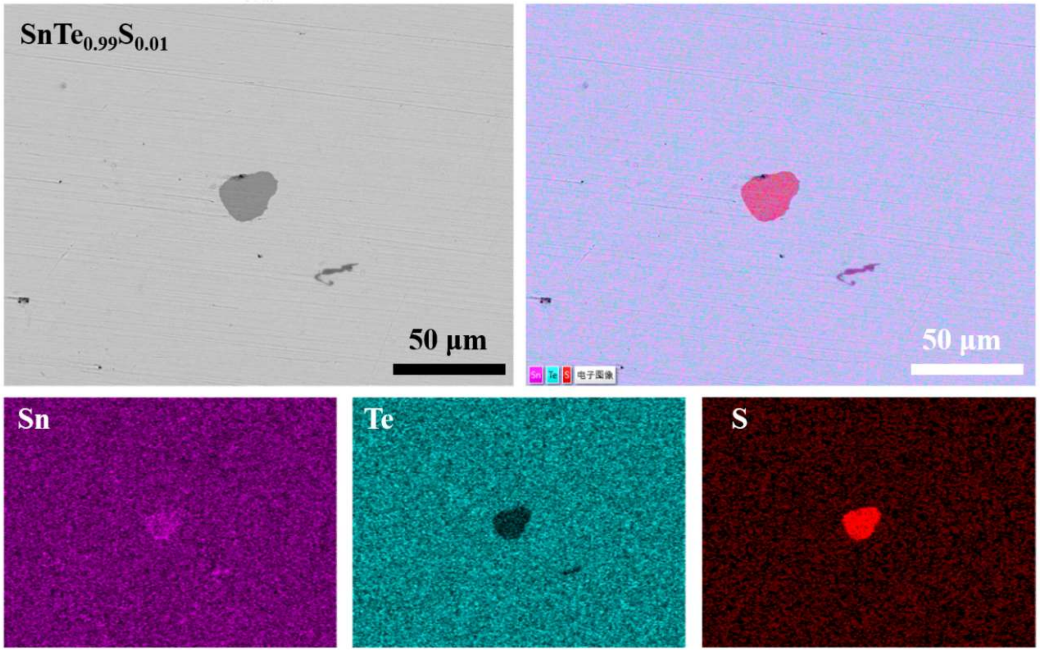

Fig. S16. BSE and EDS mapping of $SnTe_{0.99}S_{0.01}$ sample.

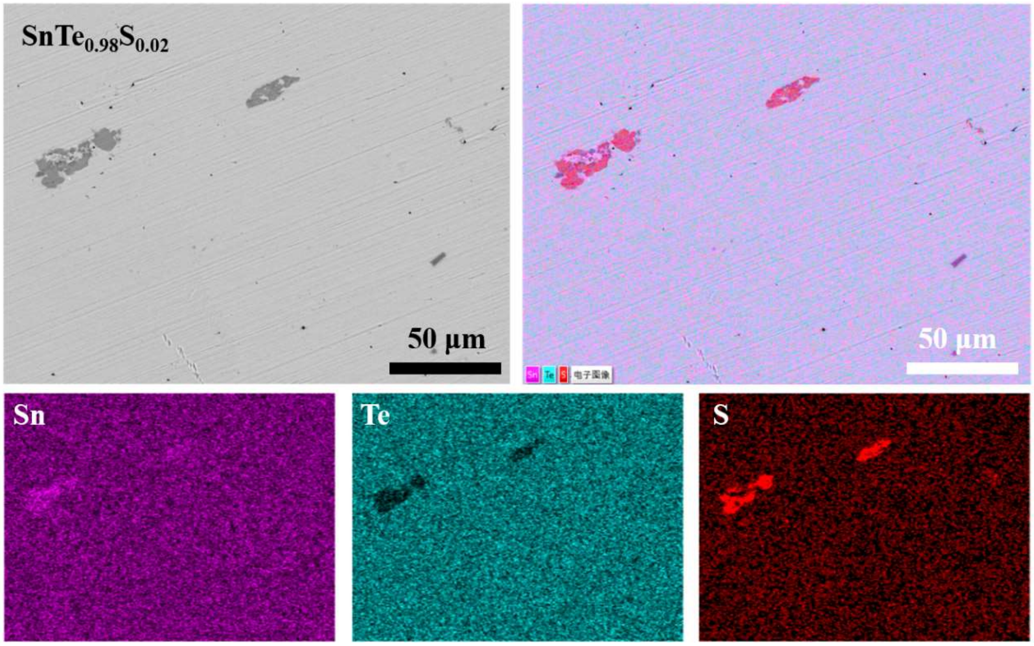

Fig. S17. BSE and EDS mapping of $SnTe_{0.98}S_{0.02}$ sample.

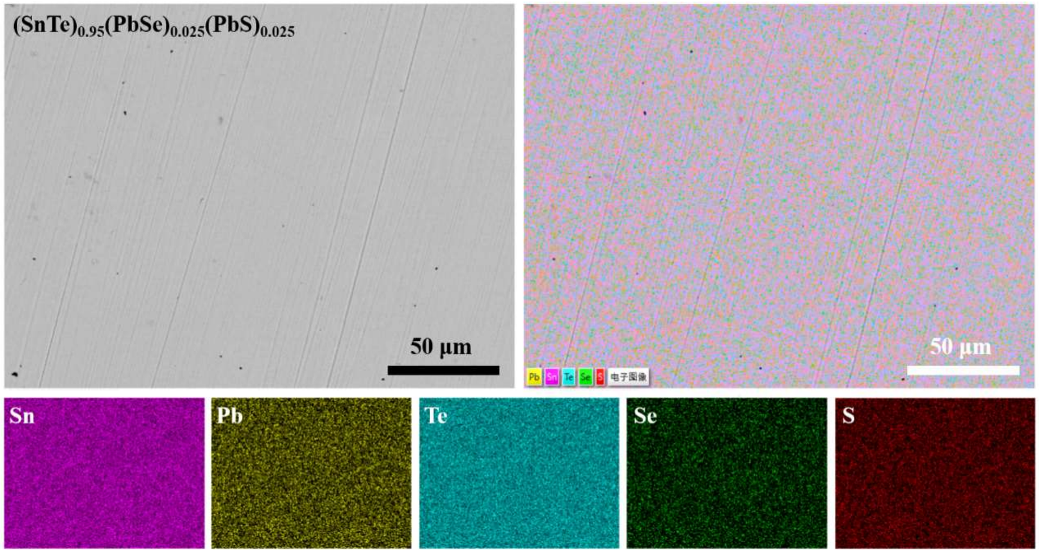

Fig. S18. BSE and EDS mapping of $(SnTe)_{0.95}(PbSe)_{0.025}(PbS)_{0.025}$ sample.

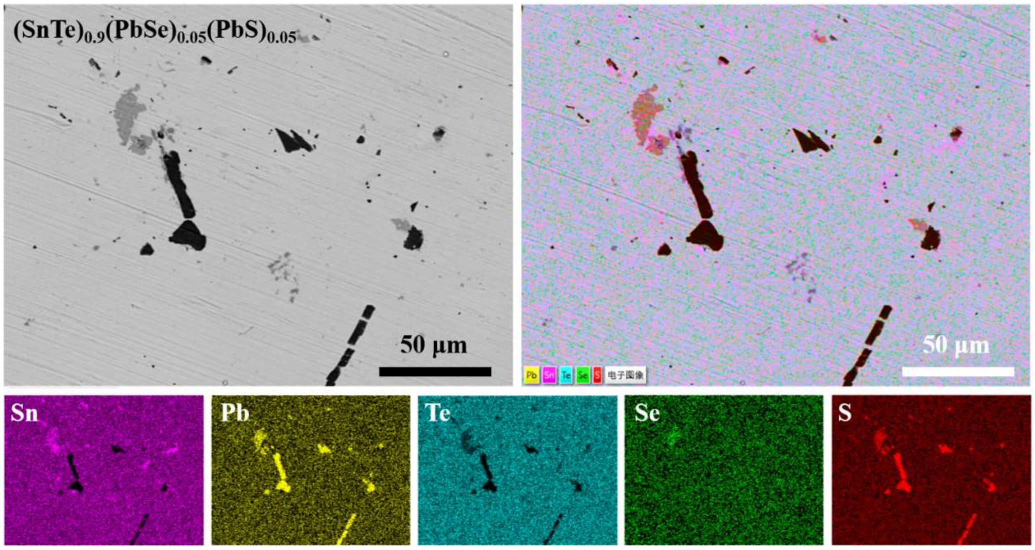

Fig. S19. BSE and EDS mapping of $(SnTe)_{0.9}(PbSe)_{0.05}(PbS)_{0.05}$ sample.

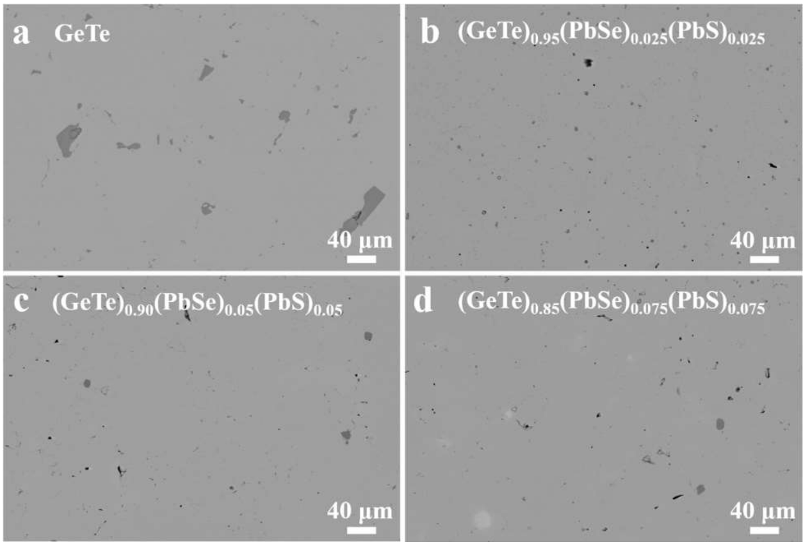

Fig. S20. Backscattering electron (BSE) images of the polished surface of **a** GeTe, **b** $(GeTe)_{0.95}(PbSe)_{0.025}(PbS)_{0.025}$, **c** $(GeTe)_{0.90}(PbSe)_{0.05}(PbS)_{0.05}$, and **d** $(GeTe)_{0.85}(PbSe)_{0.075}(PbS)_{0.075}$. Note that the dark black dots are micropores but not precipitates.

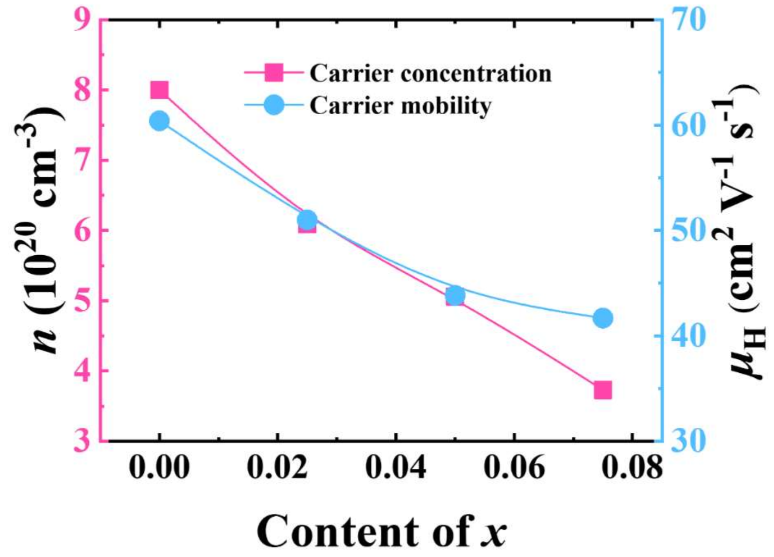

Fig. S21. Carrier concentration and carrier mobility of $(GeTe)_{1-2x}(PbSe)_x(PbS)_x$.

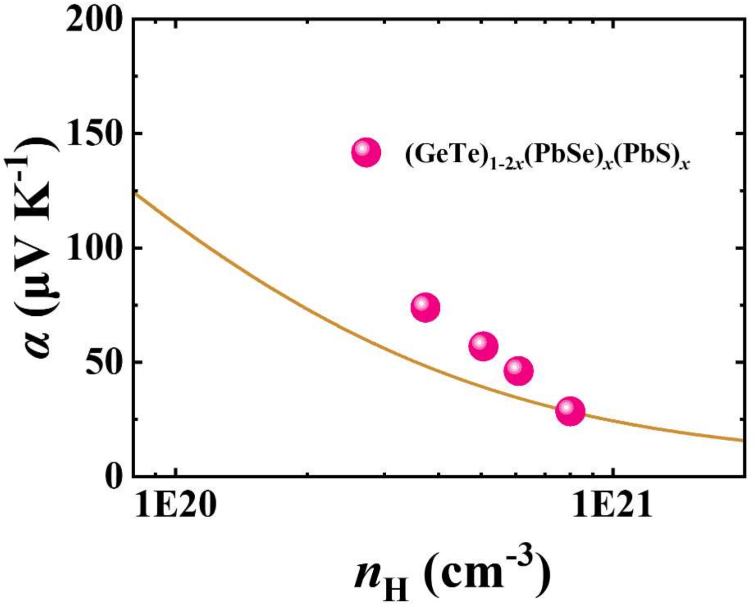

Fig. S22. The relationship between carrier concentration and Seebeck coefficient of $(GeTe)_{1-2x}(PbSe)_x(PbS)_x$.

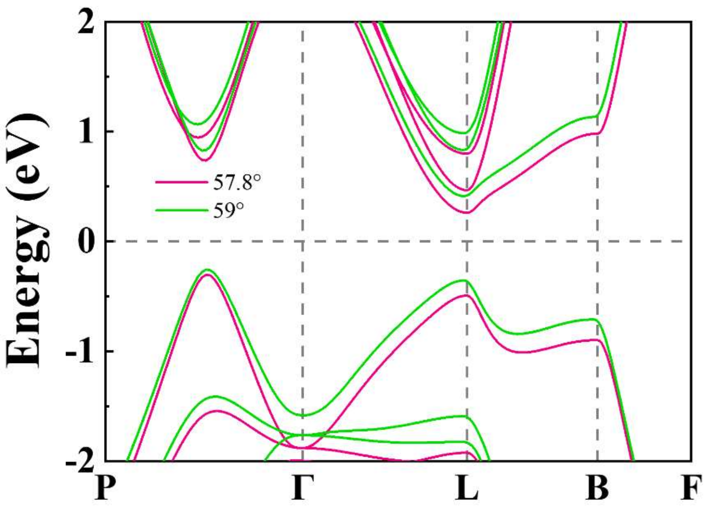

Fig. S23. Band structure of GeTe with different inter-axial angles.

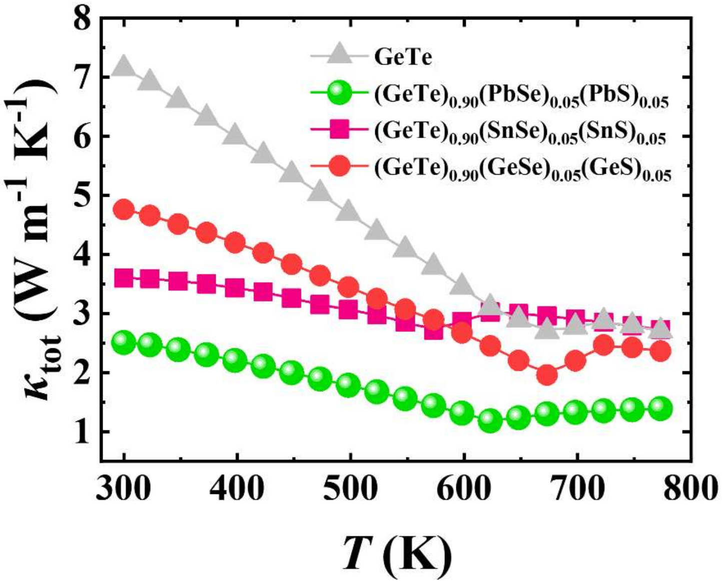

Fig. S24. Total thermal conductivity of GeTe, $(GeTe)_{0.90}(PbSe)_{0.05}(PbS)_{0.05}$, $(GeTe)_{0.90}(SnSe)_{0.05}(SnS)_{0.05}$, and $(GeTe)_{0.90}(GeSe)_{0.05}(GeS)_{0.05}$.

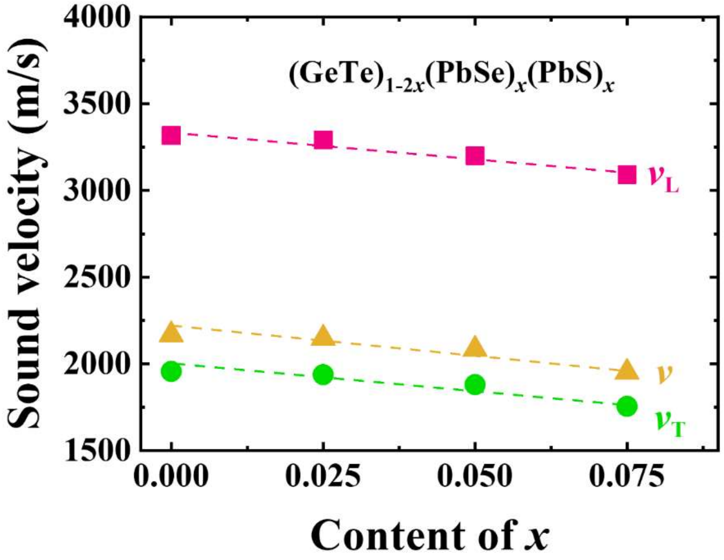

Fig. S25. The relationship between carrier concentration and Seebeck coefficient of $(GeTe)_{1-2x}(PbSe)_x(PbS)_x$.

Supplementary Note:

Thermoelectric transport properties of $(GeTe)_{1-2x}(PbSe)_x(PbS)_x$ samples

Fig. S26 displays the electrical and thermal transport properties of $(GeTe)_{1-2x}(PbSe)_x(PbS)_x$ samples. The electrical conductivity σ (**Fig. S26a**) is reduced due to the decrease of carrier concentration and carrier mobility (**Fig. S21**), and the Seebeck coefficient α (**Fig. S26b**) is increased due to the reduced carrier concentration. In addition, the α is also related to the density-of-states effective mass m^*, and the m^* is calculated based on the single parabolic band (SPB) model in the assumption of acoustic phonon scattering, as displayed in **Fig. S22**. The increased m^* may mean the change of band structure after Pb, Se or S doping. However, the first-principle calculated electronic band structure reveals that the band structures of Pb substituting Ge, Se or S substituting Te in both rhombohedral GeTe (R-GeTe) and cubic GeTe (C-GeTe) are not diverse (**Fig. S27**). In fact, the enhanced m^* originates from the inter-axial angles increasing (**Fig. S28**) induced band convergence, as shown in **Fig. S23**[2, 3]. The total thermal conductivity κ_{tot} of $(GeTe)_{1-2x}(PbSe)_x(PbS)_x$ samples is distinctly reduced (**Fig. S26d**). On one hand, the decrease of σ leads to the reduction of κ_e. On the other hand, PbSe and PbS alloying induces large mass and strain fluctuations and reduced sound velocity (**Fig. S25**) and then reduces the κ_L (**Fig. S26e**). Ultimately, the ZT value is obviously enhanced with x, as shown in **Fig. S26f**.

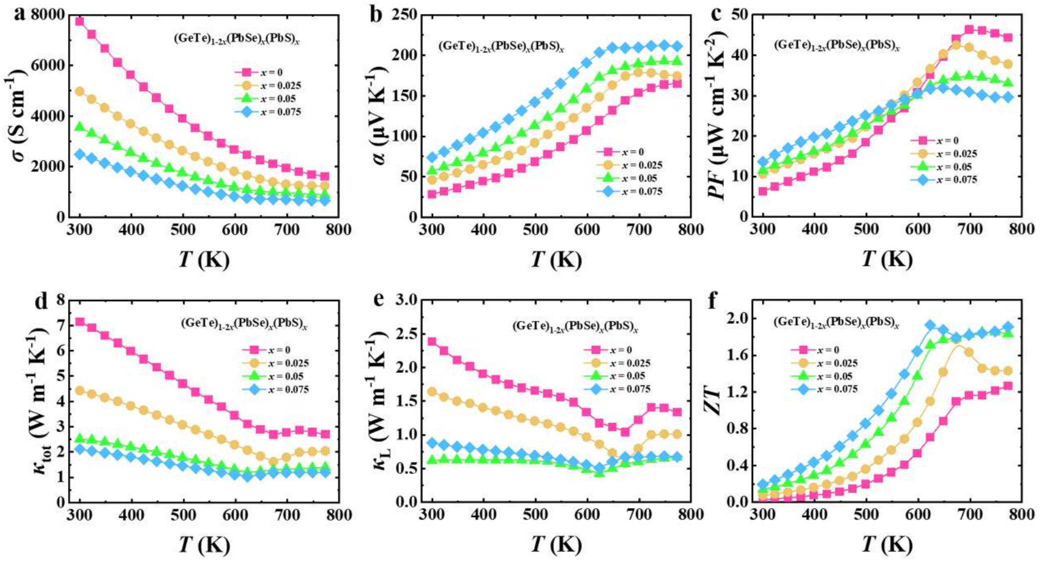

Fig. S26. Thermoelectric transport properties of $(GeTe)_{1-2x}(PbSe)_x(PbS)_x$. **a** Electrical conductivity, **b** Seebeck coefficient, **c** power factor, **d** total thermal conductivity, **e** lattice thermal conductivity, and **f** *ZT*.

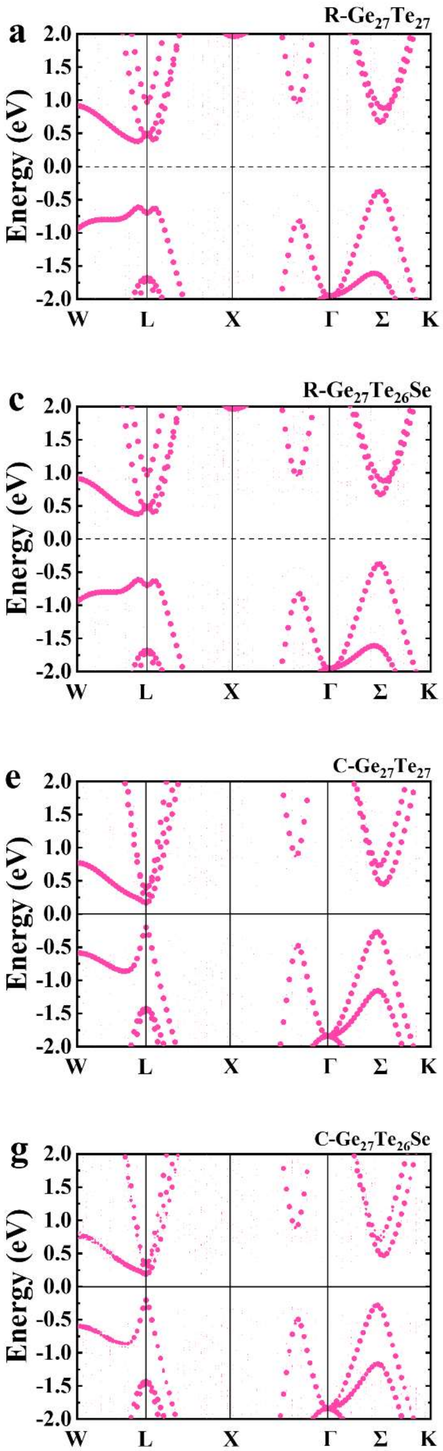

Fig. S27. Electronic band structures of **a** R-$Ge_{27}Te_{27}$, **b** R-$Ge_{26}PbTe_{27}$, **c** R-$Ge_{27}Te_{26}Se$, **d** R-$Ge_{27}Te_{26}S$, **e** C-$Ge_{27}Te_{27}$, **f** C-$Ge_{26}PbTe_{27}$, **g** C-$Ge_{27}Te_{26}Se$, and **h** C-$Ge_{27}Te_{26}S$.

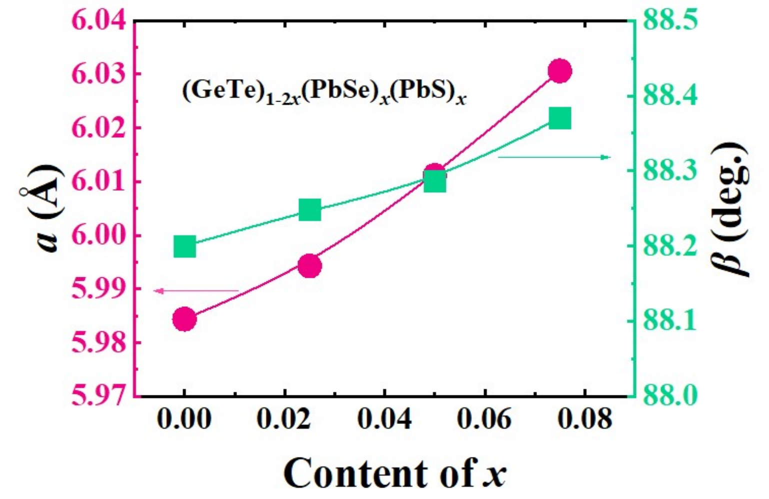

Fig. S28. Lattice parameters and inter-axial angles for $(GeTe)_{1-2x}(PbSe)_x(PbS)_x$.

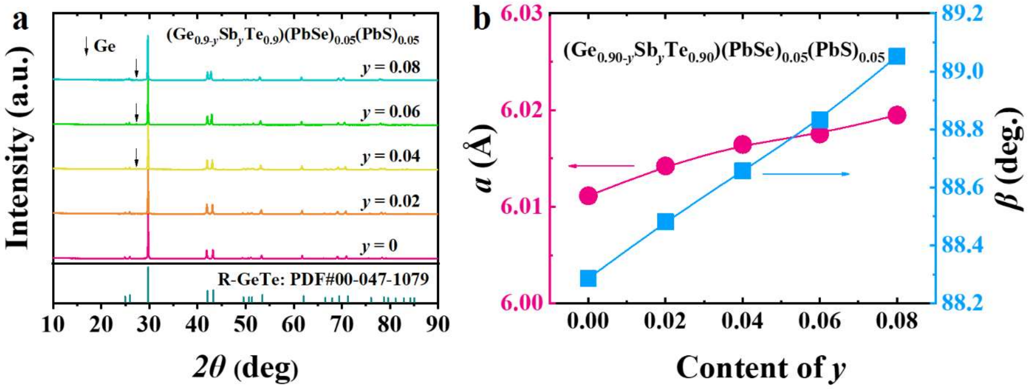

Fig. S29. **a** Powder XRD patterns, and **b** lattice parameters and interaxial angles for $(Ge_{0.9-y}Sb_yTe_{0.9})$ $(PbSe)_{0.05}(PbS)_{0.05}$.

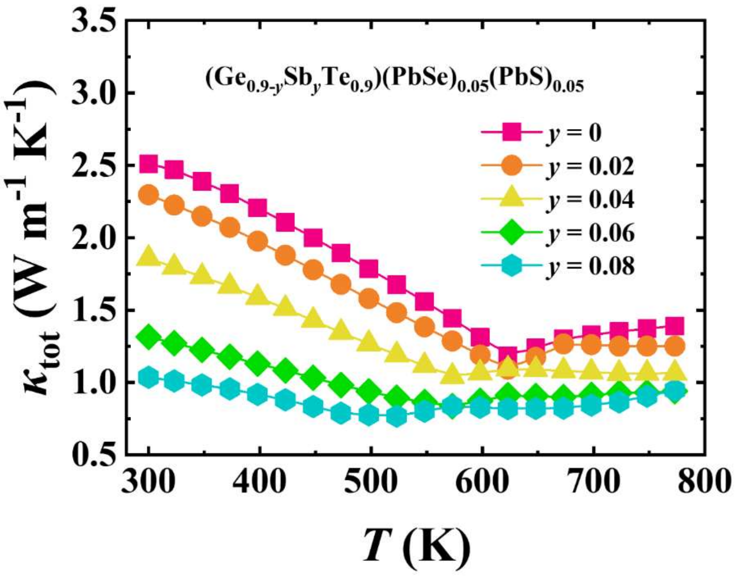

Fig. S30. Total thermal conductivity of $(Ge_{0.9\text{-}y}Sb_{y}Te_{0.9})$ $(PbSe)_{0.05}(PbS)_{0.05}$.

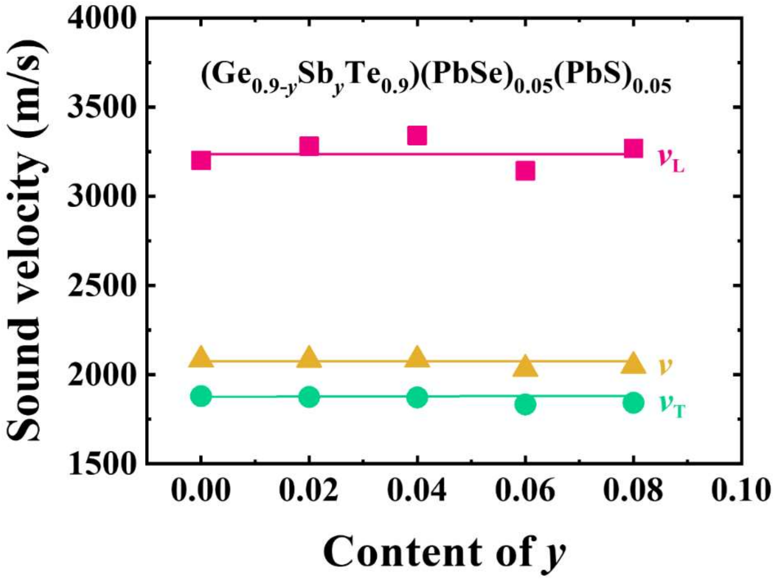

Fig. S31. Sound velocity of $(Ge_{0.9\text{-}y}Sb_yTe_{0.9})$ $(PbSe)_{0.05}(PbS)_{0.05}$.

Table S1 The elastic properties (Young's modulus *E*, Poisson ratio *r*), and the Grüneisen parameter γ is derived using eq. S(1-4) based on the measured sound velocity.

Samples	v_L (m/s)	v_T (m/s)	v (m/s)	E (GPa)	r	γ
GeTe	3292	1938	2147	56.8	0.23	1.43
$(GeTe)_{0.95}(PbSe)_{0.025}(PbS)_{0.025}$	3276	1911	2120	56.2	0.24	1.46
$(GeTe)_{0.9}(PbSe)_{0.05}(PbS)_{0.05}$	3200	1840	2043	52.6	0.25	1.51
$(GeTe)_{0.85}(PbSe)_{0.075}(PbS)_{0.075}$	3091	1755	1951	48.2	0.26	1.56

1. Jiang, B., *et al.* High-entropy-stabilized chalcogenides with high thermoelectric performance. *Science* **371**, 830-834 (2021).

2. Li, J., *et al.* Low-symmetry rhombohedral GeTe thermoelectrics. *Joule* **2**, 976-987 (2018).

3. Zhang, X., Bu, Z., Lin, S., Chen, Z., Li, W., Pei, Y. GeTe thermoelectrics. *Joule* **4**, 986-1003 (2020).
